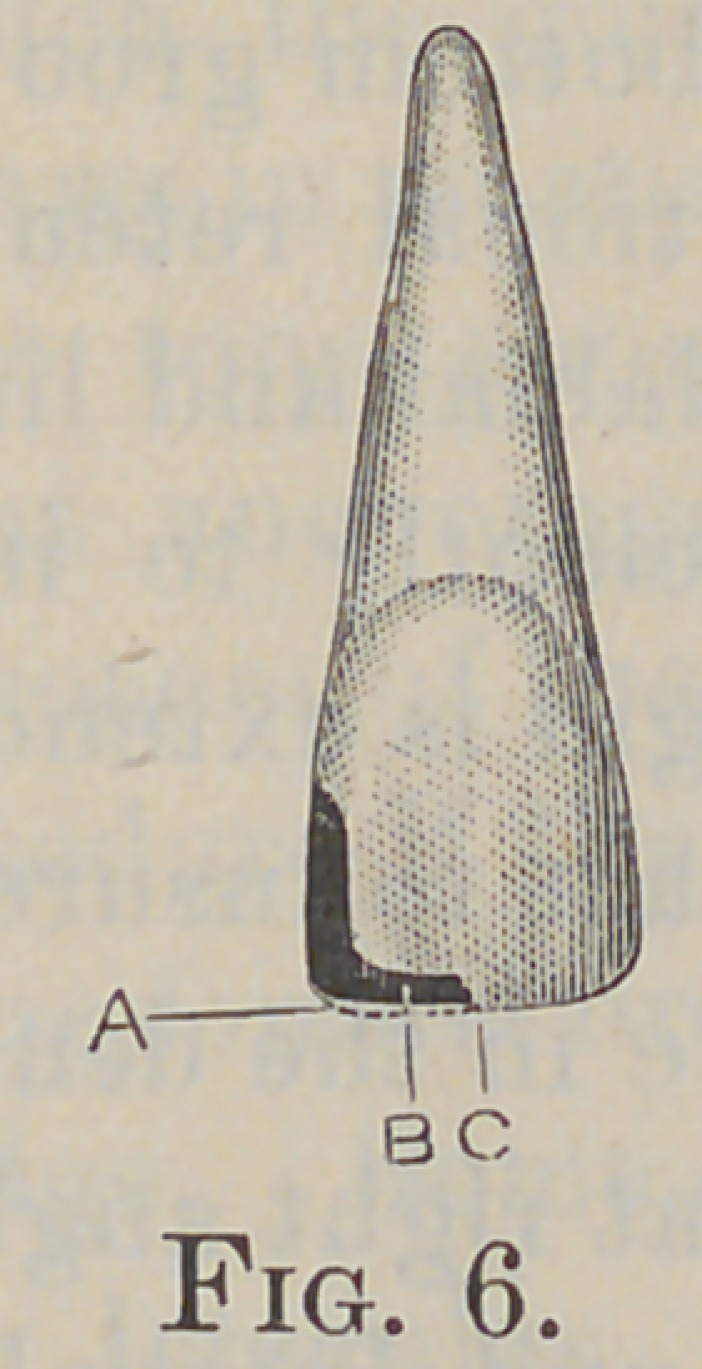# Ohio State Dental Society

**Published:** 1898-02

**Authors:** 


### THE DENTAL REGISTER.

VOL. LII.] FEBRUARY, 1898. [No. 2.

### PROCEEDINGS.

### Ohio State Dental Society.

FIRST DAY—MORNING SESSION.

The thirty-first annual session of the Ohio State Dental Society was held at the Neil House, Columbus, Ohio, commencing Tuesday, December 7, 1897.

The society was called to order at 10:30 A. M.; President L. E. Custer, of Dayton, in the chair. The minutes of the last annual meeting having been read and approved, the president read the annual address, as follows:

*Members of the Association:*

We are met for our thirty-first annual session. The work done by this society in the past and the good which it is yearly accomplishing need scarcely be brought to your notice. It has been my privilege in the past few years to be present at the meetings of the societies of a number of different States, and, with the exception of the Illinois, I have found no better organizations and no more or better work accomplished than in our own.

The objection has been raised by the dentist who has not had the advantage of a college course, that the time of the meetings is taken up by college professors with papers and theories in which they have no interest. While some may think it a loss of time to listen to the professor, this class is growing smaller and smaller. The day, we think, is not far distant when every one in our dental societies will appreciate the papers that come from our teachers and the older heads, to the extent that they will not call every one a "fine-spun theory." We should regard it a privilege rather than a burden. For my own part, and I think I speak for a great many, it is a pleasure to occasionally meet our old friends, protoplasm, the Miller theory of caries, or even a group of alcohol radicals.

The sphere of the young practitioner is one of no less importance to the society and to the profession than the older ones. It is he who in time is to take the place where now stand those who are his seniors. The beginner is not only profited by attending a dental society, but he can be a benefit to the profession from the very beginning of his career. He need not wait till he is an old man before attending a dental society. Many a young man, upon finishing his college course, enters upon the practice of dentistry with the impression that he knows it all; that there is nothing further to be learned; that there are no more worlds to conquer, and that his attendance upon a dental society would be a loss of time and energy. The student, the progressive man, however, finds delight and profit in the dental society. He regards his college education only as a stepping-stone to higher attainments. He feels that in the college he only learned the index to a great library; that he has only learned where knowledge is to be found. Such a one appreciates the good to be derived from meeting with others in the search for knowledge. Longfellow has truly said, "The mind of the scholar, if you would have it large and liberal, should come in contact with other minds."

The young practitioner may not only be benefited by what he learns from the experience of others, but, if he immediately takes part in furnishing his share of the papers presented, he will himself be benefited thereby. If you do not know anything on a subject, write an essay upon it and you will be surprised to find how much you have learned.

The importance of the young practitioner and the good which he has accomplished in the progress of dentistry has not, I think, been fully accredited. Some of the most important achievements made in dentistry have been accomplished by very young dentists, and I think it may safely be said that the majority of the substantial advances made are due to men under forty years of age. Every young man who enters the profession focuses his lens upon an object, and sooner or later the truth will be known. He comes with a new mind, and, it may be, in using a lens of lower power, sees the very thing that has obstructed the vision of those older in the profession who are working, as it were, with higher powers.

For years our State has been made the dumping-ground for many of our neighboring States. Prior to the new law, in 1892, there was only an imitation of a dental law, but the character and integrity of the examiners had a wholesome effect.

Nearly every member of this society knows the great effort that was exerted to secure even the McMakin law, and, in the face of the bad state of things resulting from it, there is little encouragement to continue the effort. But it appears from the experience of the past that if the matter is taken up systematically, by introducing a single amendment at a time, we will in time have a better law than by the introduction of a whole new bill. The frame-work of the law, as it now stands, is good enough for the purpose, and it is but a matter of correcting it in detail; and in that way, in the end, a much more satisfactory law will be the result.

There is fresh in the memory of many here a dental meeting held in Detroit some three years ago. There has not been, since the Columbian Dental Congress of 1893, such a successful and fruitful meeting of dentists anywhere in our land. The promoters were so elated with the results that it was decided to hold another, three years from that time, and the honor of entertaining the proposed meeting fell to Ohio.

The time is near at hand, and, this being the last meeting of this society before the appointed time, it will be seen that we must give some of our time to arranging for this.

Within the past year the practicability of cataphoresis has been rather fully tested, and, while there may not be the enthusiasm that was shown a year ago, it would seem that this method of obtunding hypersensitive dentin has about reached the level that it will maintain. Owing to the care, the time, and the knowledge required for making the operation a success, it will not be used in every case that presents; nor should it be.

The danger to the pulp is a matter not to be overlooked, and this should be the controlling factor in the selection of cases. It seems that if the process is carried far enough to obtund the pulp to any great extent, trouble will follow. It is almost impossible to obtund the dentin in the walls of a deep cavity, for the reason that the current, finding a better conductor in the thin layer of half-calcified dentin at the bottom of the cavity, nearly all of it flows directly into the pulp through the bottom of the cavity. The walls of a deep cavity are not usually obtunded by the current, except in a reflex manner by first obtunding the pulp through the thin layer of dentin at the bottom of the cavity. Herein lies the danger. The pulp is already trembling between life and death by the nearness to exposure, and this condition is not helped by the current of electricity which is very likely to be used in such cases, to the extent that electrolysis of the pulp is produced. In cavities where the pulp is somewhat distant, it is entirely feasible to use the current for obtunding the dentin without endangering the pulp. No one should perform the operation without using a milliameter, not any more for measuring the current flowing through the tooth than for the purpose of detecting whether the current may be leaking around the tooth and producing destructive electrolysis of the gum-tissue.

About five years ago I happened to be in Basel, Switzerland, and saw Dr. DeTrey making gold fillings with a form of gold devised by him. I was astonished at the rapidity with which he was able to build up a large filling.

Of late this gold has been introduced in this country. It appears to possess a property entirely different from any other form of gold now on the market. It is a precipitated gold, and comes in the form of mats or layers, from one-thirty-second to one-sixteenth of an inch in thickness. Under the instrument it is condensed with remarkable uniformity and density, and those who have used Watt's gold can imagine the nature of it by comparing Watt's crystal gold to a dried-out sponge and the DeTrey gold to a piece of spunk. This gold builds up very rapidly, and is especially fitted for starting fillings; for use in places difficult of access; for building up the body of very large filling, and for making a dense masticating surface. A form of gold quite similar to the DeTrey gold is that made by Hoff & McFarland, of Frankfurt, Germany. This differs from the DeTrey principally in the thickness of the layers in which it is furnished and, strange to say, the color of the finished fillings. The DeTrey gold is of a somewhat brassy hue, while the Hoff & McFarland gold is of a darker red.

Both of these forms of gold require special instruments, but the Royce pluggers will answer very well.

This year, as the program shows, a day will be given to clinics. That this is a desirable feature all will agree. An innovation due to Dr. Callahan will be tried.

Instead of demonstrating upon a patient, these operations and demonstrations will be made upon models, which can not only be seen by a great many, but can be passed around for a more minute inspection. This enables the operator to point out many things in detail which otherwise could not be seen in a clinic upon a human subject.

At the conclusion of the presidential address it was, upon motion, agreed that the election of officers for the coming year should be made the first order of business at the afternoon session, and the society adjourned to meet at 2 P. M.

### AFTERNOON SESSION.

The society was called to order at 2 P. M., and proceeded to the election of officers. The following were elected: Grant Molyneaux, Cincinnati, president; L. L. Barber, Toledo, first vice-president; H. F. Harvey, Cleveland, second vice-president; L. P. Bethel, Kent, secretary; C. I. Keeley, Hamilton, treasurer.

Dr. Henry Barnes, Cleveland, made a short address on the necessity of dental societies keeping more complete records of their members. Such things as the future members would be likely to wish to know should be made a matter of record; for instance, along with the record of every member's name and membership in the society should be a record of time and place of his birth; of his graduation; other associations, if any, of which he was a member, and official positions he may have held in societies. At the conclusion of his remarks he presented the society with a record book of his own devising, fully prepared for the purpose as he described it.

On motion of Dr. Grant Molyneaux, a vote of thanks was given Dr. Barnes for the presentation of this book, and at a later session the secretary was instructed to employ such additional help as would be needed to collect and record the facts in regard to present and former members of the society, to make the records complete.

Dr. S. D. Ruggles, of Portsmouth, read the following paper:

THE PREPARATION OF COMPOUND CAVITIES IN ANTERIOR TEETH.

The cavities to which reference is made in this paper are those of the upper incisors, in which the mesio-incisal or disto-incisal angles have either been destroyed or decay on the approximal surfaces has extended toward the incisal edge, until the enamel is deprived of strength sufficient to withstand the force of mastication (Fig. 1, A).

Fig. 1.

Fig. 2.

Fig. 3.

Before entering upon a description of their preparation, it will be well to consider for a moment the points of approximal contact and the occlusion.

In a normal occlusion the incisal edges of the lower incisors may be in contact with the lingual surfaces of the upper incisors, anywhere from the incisal edges to a point midway between them and the lingo-gingival ridges, (Fig. 2, A). In the best-formed arches the interdental spaces extend to points on the approximal surfaces which lie near the incisal edges (Fig. 3, A).

The necessity of the preservation of these interdental spaces and points of approximal contact has been urged repeatedly in recent literature, particularly in the writings of Dr. G. V. Black, until detail on this point is unnecessary.

The distribution of force upon the anterior teeth is not the same as upon either the bicuspids or molars. By this is meant that the entire force exerted in the act of biting is not transmitted in a line with the apex, but is distributed to a greater extent along the labial surfaces of the roots in their movements upward and backward as is seen in Fig. 2; the long axis of the root is not in a direct line with the force exerted. The crown of the typical incisor has an inclination of about ten or twelve degrees from the long axis of the root, and is in line with the force exerted. This fact should be taken as the principle for the preparation of these cavities. In these cavities, as in all other step or compound cavities, the object is, if possible, to have the base of the filling equal as nearly as possible the surface exposed to mastication. As an illustration, let the superior left central incisor whose mesio-incisal angle is destroyed be taken. The dam adjusted and sufficient space in which to work obtained, and all decay removed, it now becomes necessary to determine to what extent the gold must be carried over the incisal edge mesio-distally (Fig. 6, C). This will depend upon the extent of the decay on the

FIG. 4.

FIG. 5.

FIG. 6.

lingual surface. The cavity must be extended into healthy tissue, and to such an extent as to insure a sound base for filling. The depth of the step into the labial plate of enamel (Fig. 5, A), or the thickness of the gold for the protection of this plate, will depend upon the occlusion. If it be normal the fortieth of an inch will be sufficient, but in case the occlusion should be end to end with the lower incisors it must be made deeper and the body of gold heavier to withstand the extra stress.

The first step is made with a square-edged carborundum wheel, one that is fine enough to prevent chipping of the enamel. Care should be exerted to keep the step as nearly parallel as possible with the original or subsequent contour of the incisal edge, as this insures an equal distribution of the stresses; otherwise the body of gold on this surface will only be as strong as its thinnest point (Fig. 6, B).

It is now necessary to remove a portion of the lingual plate of the enamel to form the lingual step, (Fig. 5, B). This is done with a drill and chisel, or, better, with a sharp inverted cone in the engine. The extent of this step also depends on the extent of the decay, but a firm base in healthy dentin is necessary (Fig. 4, D). Its floor is formed at a right angle to a line extending from the incisal edge to the junction of the enamel and cementum on the labial surface, which line is approximately the long axis of the crown; and, mesio-distally, it should be parallel with the incisal edge (Fig. 5, D).

In preparing this step care should be taken that the labial plate of enamel be not robbed of all its dentin support, else the gold will become visible through this plate. If possible a slight flat-bottom groove should be made in the dentin at this point for additional retention (Fig. 4, A). All overhanging enamel on the labial and lingual surfaces unsupported by dentin is removed sufficiently to insure self-cleansing margins, and the gingival margin is extended far enough beyond the point of approximate contact to insure its cleanliness (Fig. 3, B). A flat seat is now made in the dentin at the gingival portion of the cavity (Fig. 4, C), at right angles to the axial wall (Fig. 5, C), extended labially and lingually as the case permits, making at the same time undercuts in the dentin, as shown in Fig. 4, B. and E.

A flat-bottom retaining-pit is now made in the labial and lingual extremities of the gingival base, diverging from each other (Fig. 4, F); also one in the lingual step, at the junction of the pulp and axial walls (Fig. 5, E). All retaining-pits should be made in dentin. In preparing the margins a thin, sharp, flexible chisel should be used, giving them a bevel of fifteen or twenty degrees, care being taken to obliterate all sharp angles, as curves are much more pleasing to the eye.

DISCUSSION.

Dr. C. R. Butler, Cleveland, said that he had an opportunity to look over the paper and photographs of the drawings, and was struck with the evident care and study given by the author in the preparation of the paper. All questions have two phases, the ideal and the practical. It is well to have high ideals, for then the results of our efforts are likely to be more satisfactory and complete. Unless an artist has a perfect ideal of what his picture or the piece of sculpture which he is essaying should be, his hand, no matter how cunning and skillful, will never be able to produce a fine and worthy production.

As dentists we know there are no two cases of just the same arrangement of the teeth, either in form or structure. The ideal treatment of compound cavities in anterior teeth will depend largely upon the state of the remaining teeth. We must remember that the force brought against the filling, if direct, is very liable to dislodge it. The author says the gold must be one-fortieth of an inch in thickness, but even if it were one-tenth or one-eighth of an inch thick the occlusal force of the antagonizing teeth, if acting directly on the filling, will stretch the gold and loosen it. The aggregate force exerted by the jaws in mastication during twenty-four hours will reach over two tons, enough to stretch almost any substance. No matter what anchorage you have, the force will be applied just at the distal corner of the gold. The author mentions the need of different procedure if the teeth are regular or irregular; in irregular teeth undue force is brought to bear upon particular portions of the cutting-edge. Some of the worst cavities we have to repair are in these irregular teeth, where you can not get at them properly. In his opinion, it is advisable to save as much of the tooth-substance as possible, as the natural edge will not stretch under the force of use as even a heavy gold edge will. It is often better to sacrifice the esthetic appearance to durability.

There can be no successful practice based upon arbitrary rules and formulas, but a rule must be made for each individual case. A high ideal and carefully-thought-out theory of how to treat the cases in the best manner will, however, be a great help; but each operation will have to be adapted to the necessities of the case, always remembering that strength of the operation and durability are the all-important aims.

Dr. Ruggles said his drawings had been made from teeth illustrating exactly the course of the work. He felt that he had not spoken sufficiently of the necessity of avoiding approximal contact. It is very important to avoid this in anterior fillings. The force brought to bear in such cases is very liable to produce such a change of the gold as to cause overhanging margins.

Dr. J. B. Beauman, Columbus, said he had been doing this class of work for forty years. At a convention held at the White Sulphur Springs Dr. Atkinson filled a tooth, using the same method, and that was where he got the idea. In the case of a central tooth, when the cavity does not extend quite to the cutting-edge, the corner of the tooth must be cut away, as the gold filling is stronger than the thin shell of the enamel would be if left to bear the strain.

Dr. Ruggles, in closing the discussion, said that a few words on the finishing of the filling may be important. There is a natural desire to restore the original outline of the tooth, but it is better to have the lingual surface an inclined plane, because then the food will slide over it and less force will be brought to bear on the filling than if the natural shape of the tooth is restored.

The subject was passed, and Dr. Charles A. Hawley, Columbus, read the following paper:

SECONDARY, OR STORAGE, BATTERIES FOR CATAPHORESIS.

What is a storage battery; how does it act, and what is its especial fitness for cataphoresis? are the questions we wish to consider in this paper, and it is the belief that the better we understand the construction or action of an appliance of any kind the more intelligent and successful will be our use of it that prompts the presentation of the subject for discussion here.

The history of the development of the storage battery goes back over a period of nearly forty years. Many attempts to store electricity by means of its action on various substances had previously been made, but Plante, a Frenchman, discovered the secret, the adaptability of lead for the purpose. He immersed two lead plates in dilute sulphuric acid, and connected one to the positive, the other to the negative, pole of a primary battery. A slight chemical change was produced between the plates and the electrolyte, and when they were connected by a wire an electric current was produced. They were then connected in the reverse order and charged and discharged, increasing the current each time, and as the chemical change became greater the discharge of current also increased. This process was continued for several months, with the final result of producing a "cell" which, when charged, had a thick coating of lead dioxid on the positive plate and a spongy lead on the negative; but when discharged had a coating of lead sulphate on both. During the discharge a very powerful current was produced, sufficient to melt iron rods. Though in the present improved storage batteries the style of cell and the process of forming are much changed, the principle is the same—*i. e.*, the electricity is stored or accumulated in the form of an unstable chemical compound. The peculiar and valuable property of this compound is that its chemical energy can at any time be reconverted into electrical energy by connecting the two plates.

The current produced in the complete discharge is practically equal to that used in producing the chemical change in storing. In winding a clock we store the energy of motion by the tension of a steel spring; in the storage cell we might compare the action of the chemical compound to the tension of the clock spring, and electricity with the energy of motion, or the muscular power. This action is entirely different from that of a primary battery. In the primary battery the chemical disintegration of one of the plates by the action of the fluid produces the current, but this process can never be reversed.

A number of changes have been made in the form of storage batteries, leading up to the greatly improved and nearly perfect ones in use now. When the exact nature of the chemical compound on the plates was found it was but a step to coat lead plates with lead oxid and sulphuric acid, which, by a few days' charging, could be changed to lead dioxid on the positive and spongy lead on the negative plate, thus saving the months of time in forming. Plates have also been made in the form of grids, with the active material, in the condition of paste, placed in the holes. The trouble with this form of plate has been found to be in the bending or buckling during discharge, which is caused by the great expansion of the paste. The paste is also apt to scale off the plates or fall out of the grids and, accumulating at the bottom, short-circuit the cell. Batteries are now on the market which claim to overcome completely these objections.

The especial fitness of storage batteries for cataphoresis lies in their constant and steady voltage during discharge, which is due to several causes: First, the internal resistance of the cell is very low, owing to the small space necessary between the plates. With perhaps a few exceptions, the internal resistance of primary batteries is very high, and interferes largely with their efficiency. Second, there is no such thing as polarization of a storage cell. Polarization unfits most primary cells for closed circuit work, or for holding a steady current for any length of time. The attempt to overcome it has led more than anything else to the great number and variety of cells on the market, and the number in itself is an evidence of failure. In comparison with the Edison direct current, the storage battery presents several advantages which we will mention briefly: First, freedom from the influence of induction currents along the line, or other causes which produce variation of voltage. Second, absence of danger during storms, accidents at power station, or ground connection by way of the fountain spittoon. Third, it obviates the trouble of reducing the unnecessarily high voltage of the Edison current with a controller.

The character and rate of discharge of a storage differs considerably from that of a primary battery, in that a very much larger volume of current can be obtained by decreasing the resistance in the external circuit. The capacity of a storage battery is measured in ampere hours, or the number of hours the battery will, when fully charged, discharge one ampere current before becoming exhausted. But the discharge can be completely controlled by varying the resistance in the external circuit. For example, a ten-ampere battery will discharge one ampere for ten hours, or, reducing the external resistance, ten amperes for one hour, or, reducing still further, twenty amperes for one-half hour, forty amperes for one-fourth hour, etc. If used with very great external resistance, as in cataphoresis, it would discharge a current of say five milliamperes for two thousand hours. At this rate, if used one hour a day every day in the year, a ten-ampere battery would last between five and six years before becoming exhausted. No such amount of current can be obtained from a primary battery of equal voltage. We might here again compare the storage battery in its discharge to the action of a clock spring, which, when wound, can be released at once by a sudden spring or by a steady motion for a long time. The batteries can be charged from almost any source of current, provided the voltage is at least ten percent higher than that of the battery, as otherwise the action would be reversed. The use of a battery for five or six years, as alluded to above, without charging would probably not be practical, as manufacturers claim that to keep them in best condition current should be added much oftener.

In practical results in cataphoresis, I have found that the storage battery carries out all that would be expected of it from theoretical consideration. After using the dry-cell battery and Edison direct current with various shunt controllers, I have had much better success with the storage battery with a series controller. The application is smooth and painless, and the pain limit can be very closely followed in increasing the current without signs of fluctuation. In other words, my experience seems to indicate that in cataphoresis the source of the current is as important a factor as the form of controller.

DISCUSSION.

WM. H. HERSH, Piqua: He was opposed to the use of the storage battery for many reasons. The storage cell made up of lead plate immersed in sulphuric acid is not capable of originating any current in itself, but gives off current which it receives from some outside source. If it is left idle for any considerable time it will become sulphated and its strength will be lost. It must be used nearly constantly to give the best results, and the best cells only give out ninety percent of the current which has been put into them. Most cells will not give higher than from fifty to sixty percent. The constancy in voltage is no better than the sal-ammoniac cells, and bichromatic cells, or dry cells, are just as regular. The only case in which the storage battery is to be preferred is when you are using a shunt instrument of low voltage. His experience had taught him that storage cells were very dirty, and objectionable on this account. When compared with the 110-volt current, the storage cell is superior, but there is no occasion to use the 110-volt current, nor should a battery of high capacity be used when all that is needed is from two to five amperes.

Dr. W. A. Price, Cleveland, commended the paper highly, but was disappointed that it did not go into detail more thoroughly as to the bulk and price of the batteries and expense of maintaining them. For cataphoric uses the cell should be of very low amperage. If we had a cell, such as one firm had tried to get up, of one ampere it would be very satisfactory, but it is questionable whether we would get a more uniform current than from a primary cell. He had used dry cells for a year; these only cost twenty-five cents each, and seemed as good at the end of the year as at the beginning. He thought the ideal apparatus for furnishing current for cataphoric purposes was a little water-power dynamo which would run by the power of water from the spigot, and utilize these for charging storage batteries, using small batteries and charging them frequently. A fifty-ampere hour storage battery would cost fourteen dollars each, while the dry cells cost only twenty-five cents each. They should be used in series and so arranged as to get a very gradual increase. He asked how much current those present used.

Dr. L. E. Custer said he used about three-tenths of an ampere.

Dr. Price said there was no milliampere meter made that gave us small enough graduations, nor is there a good controller in the market for cataphoresis.

Dr. W. H. Todd, Columbus, asked if a milliameter could be made to register low enough to work in series, so that it would not affect the cataphoric operations.

Dr. Custer said it would not affect it at all; you would add more voltage.

Dr. Price said the lower the sensitiveness the less would be the resistance, and the less the resistance was the more sensitive would be the instrument.

Dr. Hersh said he used as a maximum current from two to five milliamperes. He does not mean this on teeth that are alive, but for relief of sensitive dentin. His milliameter will register about the same as others. He had constructed one whose needle moved about seven inches for one milliampere, but he found that it was sensitive to outside influences, and has been told that all such would of necessity be so.

Dr. Hawley said that a dry-cell battery when new was efficient, but when about half used up there would be much fluctuation in the current. If there were no fluctuation in the current there would be no necessity for shunt controllers. He believed that a storage battery could be maintained in good condition with the use a dentist would give it in his work, and they are not expensive. He spoke of the Willard concentric cell battery, which he said cost fifteen dollars and it was not dirty. The controller is a series controller, and it will not give success with dry cells. The Edison current will show fluctuation of voltage. In one test which he made a Mescot dry cell lost in thirty minutes about thirty percent of its strength, while a storage battery began with two and one-tenth and left off with one and ninety-seven-hundredths.

The subject was passed, and the society adjourned till next day at 9:30 A.M.

### SECOND DAY—MORNING SESSION.

Dr. W. T. McLean, Cincinnati, read the following paper:

### A METHOD OF TREATMENT TO PROMOTE THE UTILITY OF PULPLESS TEETH.

This is one of the various important pathological conditions in which every dentist is more or less interested, owing no doubt to the fact that there are so many necrotic pulps found in the mouths of patients who present themselves to dentists for relief and treatment. It is a pleasure for me upon this occasion to present to the members of this society a method of treatment which has been productive of satisfactory results in my practice. It is well understood that the tooth receives its principal nourishment from the pulp, and if it were not necessary for its preservation there no doubt would have been a natural factor to eliminate it. It is known that after the age of twenty-five there is an improved calcific change occurring in the structure of the tooth, and this improvement is greatly lessened when the pulp is destroyed. Owing to the fact that there is organic and inorganic matter which enter into the formation and substance of a tooth, they are equally essential for its maintenance. I divide pulpless teeth into two major classes—viz, the medico-surgical and the septo-carious. The first class are those where the pulp is found exposed, vital or partially so, and the second class are those where the pulp has become devitalized by septic and carious encroachment. In the first class the treatment is not complicated and the prognosis is good. In the second class there is invariably a septic condition present at the apices of the roots and frequently a necrotic condition of the pericementum, and possibly a disintegration of the alveolar process, the result of necrosis. In this class the prognosis is only fair. I never attempt to treat a pulpless tooth upon which I can not apply the rubber-dam; to my mind this is imperative; after its accomplishment I obtain direct access to the cavity and canals of the tooth to be treated, never fearing the sacrifice of enamel and dentin sufficiently to that end. In the first class the removal of the vital pulp is painlessly accomplished by the chloride of ethyl spray (Bengue), which is supplied by Leeming & Co., New York. This spray requires about ten minutes' time; after the removal of the pulp, which is done with a stiff barbed broach, the tooth is permitted to return to its previous condition; after which the thorough cleansing and dehydration of the dentin surrounding the canal is accomplished. The canal is well wiped with Ceylon oil of cinnamon and hot air blown into it for a few minutes; the heat vaporizes the medicament and causes it to permeate the dentin slightly, and renders the canals and apical space sufficiently aseptic to prevent sepsis. The canals are now filled with powdered asbestos made into a thin paste with a fifty percent solution of silver nitrate; this paste is pumped into the canals by using a plain broach upon which is wrapped a few fibers of cotton, and completely filled. Harvard cement is used to protect the contents of canals. The tooth thus treated is given forty-eight to ninety-six hours to become accustomed to its changed condition, after which filling, crowning, or preparation for bridge abutment may be accomplished with propriety.

In the second class the operative precedure differs, inasmuch as complete asepsis is more difficult to obtain, and a correct diagnosis can not be made as the degree of the pathological condition can not with exactness be ascertained. We find in this variety foreign matter, pus, and possibly necrosis of the alveolus. The treatment is as follows: The rubber-dam is applied, the cavity and canals are opened, free access is obtained with aseptic instruments, and hot water is injected into canals by a hypodermic syringe until all loosened *debris* is removed. Next pyrozone is injected; if pus be present it will be forced out; a few injections will suffice. Bibulous paper placed conveniently will catch and absorb the overflow of medicament, and prevent soiling of the patient's clothing. The canal is next dehydrated with Evans' root dryer, and cotton saturated with oil of cinnamon loosely placed; and retain in place with gutta-percha stopping. The patient is dismissed with instructions to return in forty-eight hours. The treatment is continued for three or four sittings, depending upon the severity of the case and the recuperative power of the patient, allowing the same length of time between each treatment. When a fistulous opening is present and the exudation of pus is noticeable, I burr through this opening and endeavor to reach the apical space and inject pyrozone through the canals and have it traverse the fistulous tract. The object is to mechanically and aseptically cleanse it. I next partially dry this tract, and with the hypodermic syringe inject oil of cinnamon so that it oozes from the gum-opening. This is continued until pus formation, fetid odor, and sensitiveness are eradicated. The completion is the same as the first class, using my judgment in performing the most desirable permanent operation.

Dr. J. Taft, Cincinnati, said the subject of the paper is an important one, because exposure of pulps and the death of pulps are so frequent that it is desirable that all dentists should have a clear knowledge of the best way to treat the cases when they are found in his practice. No one has practiced long without meeting many cases of pulpless teeth and those having exposed living pulps. Proper treatment will often result in a prolonged retention of pulpless teeth, making them serviceable organs. There is a great variety of treatment both of pulpless teeth and those having exposed pulps. The paper gives the impression that all exposed pulps should be destroyed, but he thought the retention of the pulp, when at all possible, was very important, as the dentin is living matter and its continued life depends upon the preservation of the pulp. When this is destroyed the dentin becomes a dead tissue. The paper is so worded as to leave us to understand that when the pulp is dead the nutrition of the tooth is diminished, but in a great proportion of cases the nutrition of the dentin is absolutely cut off on the death of the pulp.

In the great majority of cases, when the pulp is recently exposed and only by a small orifice, its vitality can be preserved. I know many think that all exposed pulps should be destroyed. The feeling is that to take it away makes an end of the trouble; but does it end it? Is it not true that pericementitis and alveolar abscess will often follow destruction of the pulp?

When the pulp must be destroyed the course recommended by the paper is much better than to poison the pulp by the use of arsenious acid. This is often the cause of trouble afterward; sometimes the trouble follows quickly; sometimes it appears long afterward. This can be demonstrated by any one who carefully notes the results. There are many ways of removing pulps without pain; by the use of cocaine, for instance, in crystals or in solutions applied to the pulp; or it may be injected into the pulp, which may then be removed. Cataphoresis also will anesthetize it in almost every case, so that the pulp may be removed with as little pain as you would remove a shred of gum-tissue from the margin of the gum. While the dentist has these agents at command, it is almost, if not quite, criminal for him to use so dangerous a method as poisoning with arsenious acid.

The paper did not make any reference to the variety of conditions found in pulpless teeth. Sometimes the pulp is found to have been mummified by natural processes; there is no odor, nothing left but merely the skeleton of the pulp. Other times the pulp is in a more or less decomposed condition; and it may be without odor but still be an active irritant on the tissues beyond the apex of the root. More frequently the state of the tooth is one of very foul odor, and the tissues beyond are suffering acutely. If a pulp is mummified and dried, it may be removed with scarcely any antiseptic treatment, but measures to make the canal aseptic become necessary when there is any odor of decomposition. In some cases the pulp may be very offensive and still make no impress on the tissues beyond; sometimes because the apex is so closed that there is no communication, or it may be that the system is so vigorous that the effect is overcome by Nature's methods of disposing of irritants. The latter cases are very favorable, as when the pulp is removed you need dread no after effects. In many cases, probably, antiseptic treatment is not necessary, but still it is always advisable. Of the vast variety of antiseptics employed, most are efficient. Sulphuric acid is good; silver nitrate is also efficient, but some others are just as good and will not discolor the tooth as silver nitrate will. Mixing with asbestos is a new idea, though the advantage of it is not quite clear, except that the asbestos is indestructible. Silk or flax would answer as well. Fully saturated with silver nitrate, they would not undergo any change in the tooth-canal.

The subject was passed.

*( To be Continued. )*

A WASH of equal parts of glycerine and lactic acid will remove moth patches and freckles from the face.

## Figures and Tables

**Fig. 1. f1:**
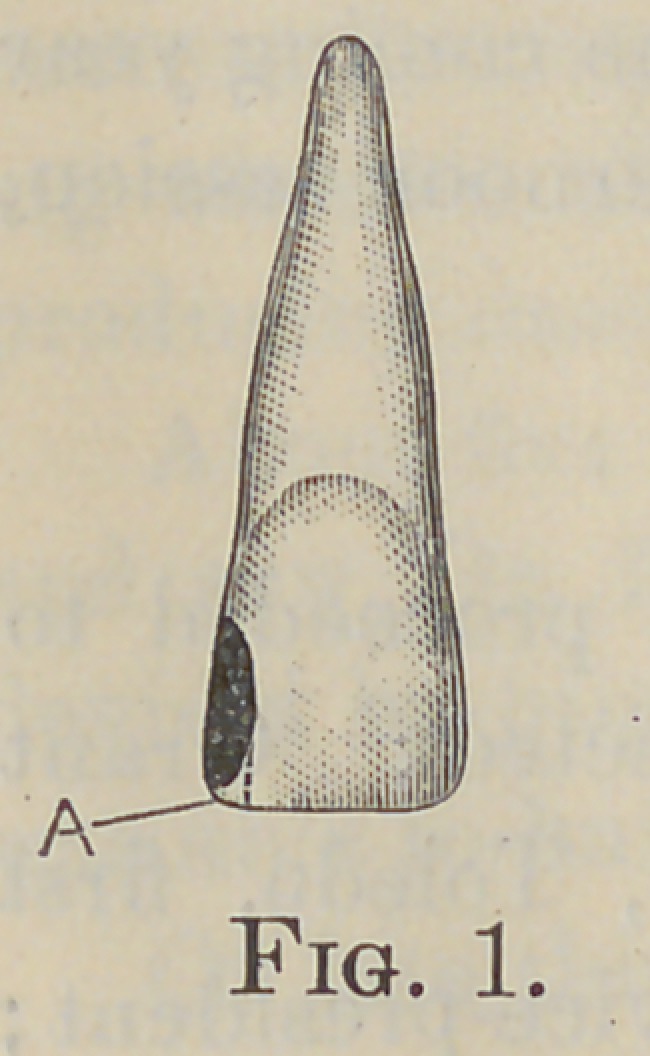


**Fig. 2. f2:**
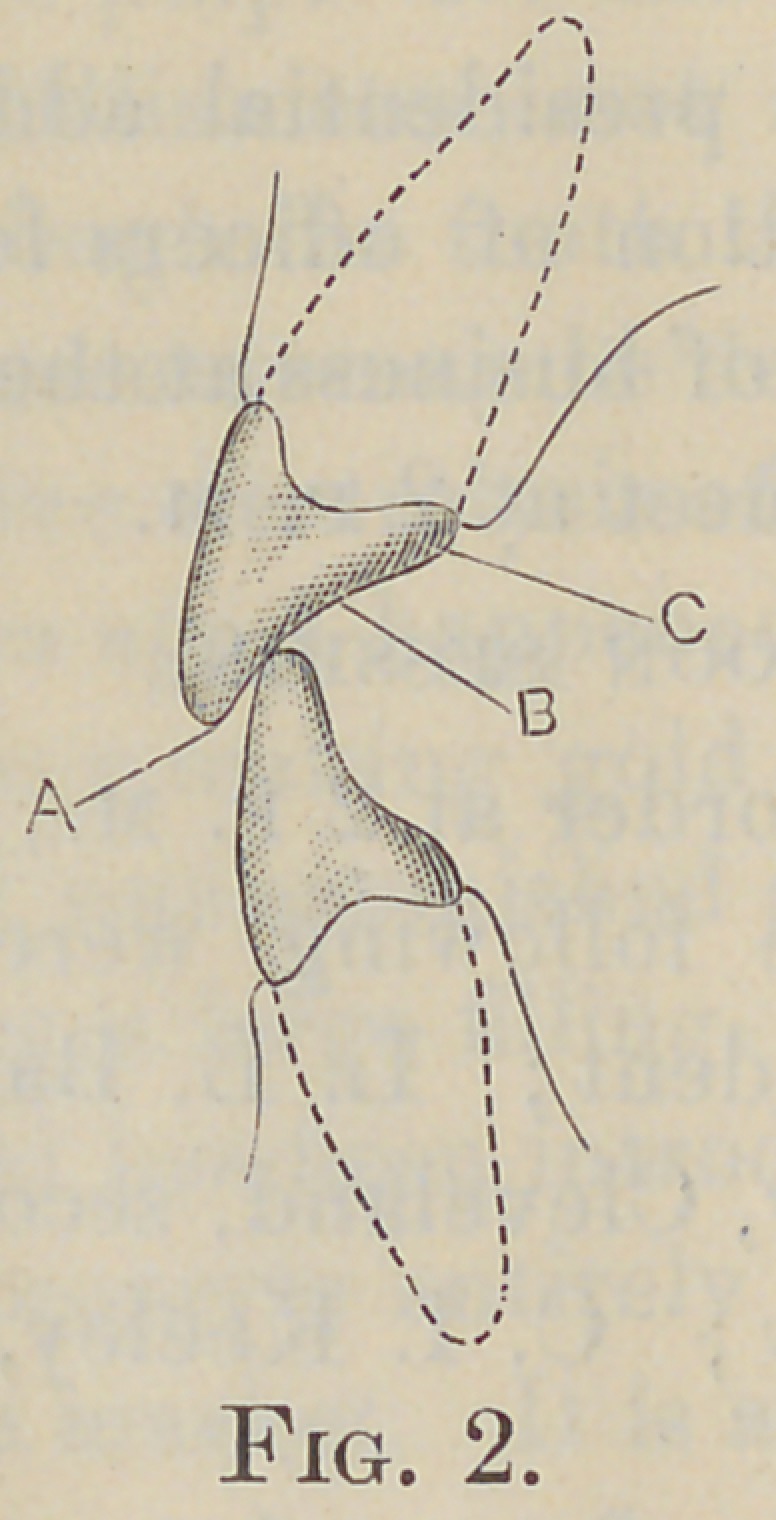


**Fig. 3. f3:**
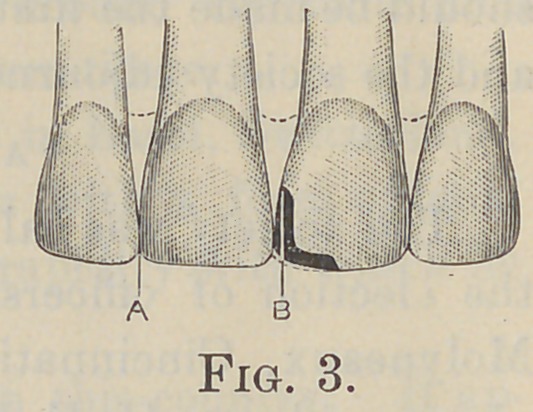


**Fig. 4. f4:**
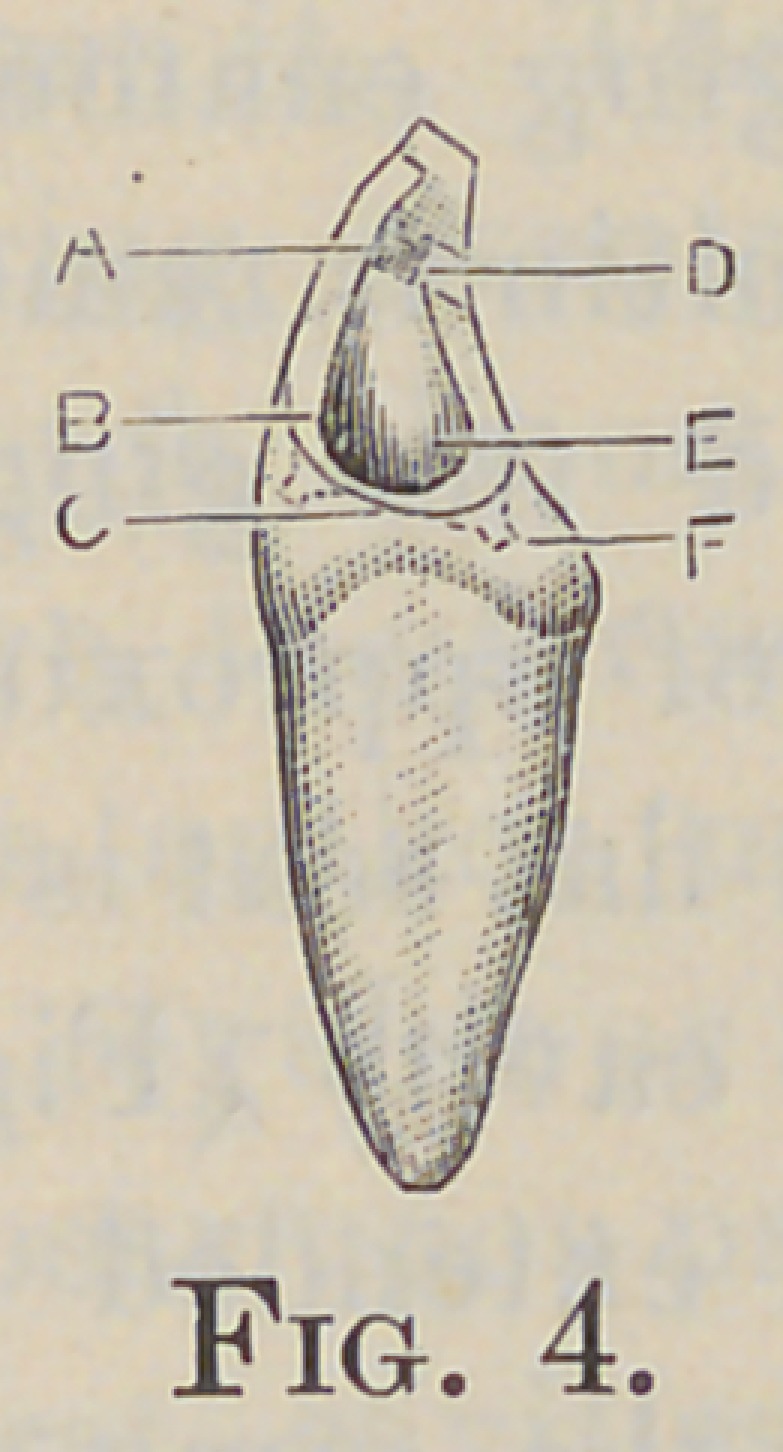


**Fig. 5. f5:**
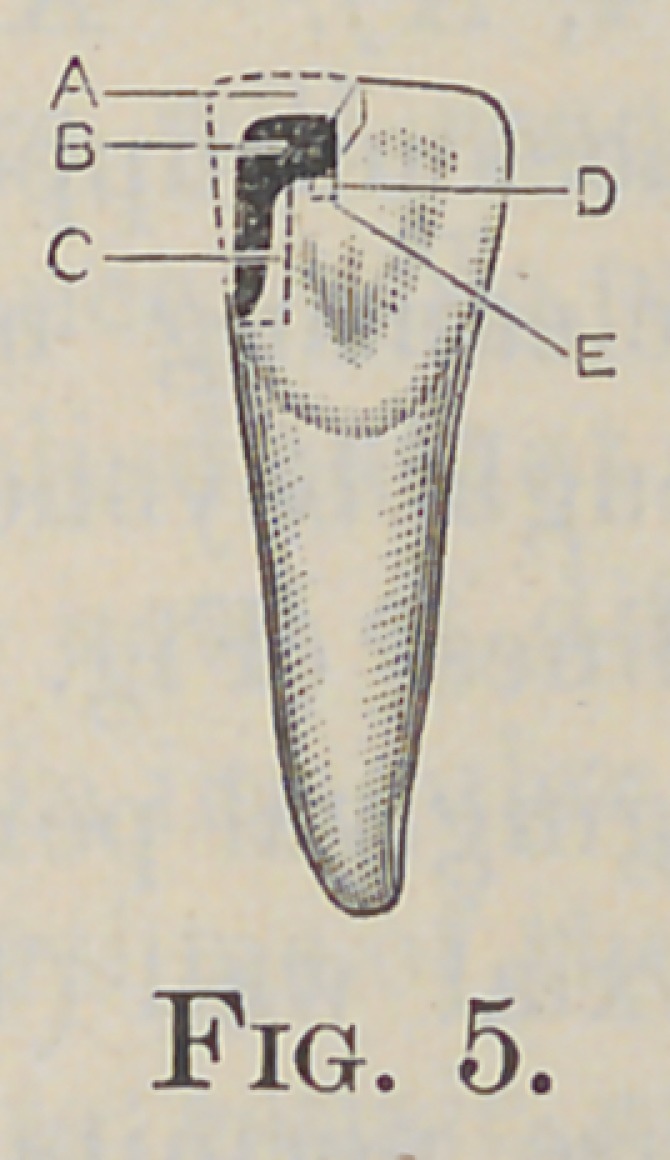


**Fig. 6. f6:**