# Correction

**Published:** 1898-04

**Authors:** 


### Correction.

In the February number, page 60, second paragraph from the top, latter part of paragraph should read: He felt that he had not spoken sufficiently of the necessity of restoring the proximate contact. This is very important.

